# Control of crystallization and magnetic properties of CoFeB by boron concentration

**DOI:** 10.1038/s41598-022-08407-6

**Published:** 2022-03-16

**Authors:** Jun-Su Kim, Gukcheon Kim, Jinwon Jung, Kuyoul Jung, Jaehun Cho, Woo-Yeong Kim, Chun-Yeol You

**Affiliations:** 1 Department of Physics and Chemistry, DGIST, Daegu, Republic of Korea; 2 SK Hynix Inc., Icheon, Republic of Korea; 3 Convergence Research Institute, DGIST, Daegu, Republic of Korea; 4 Department of Materials Science and Engineering, Korea University, Seoul, Republic of Korea

**Keywords:** Materials science, Physics

## Abstract

Controlling the crystallinity of CoFeB is the most essential issue for designing various spintronics devices. Here we show the microstructure and magnetic properties of MgO/CoFeB/MgO structures for various boron concentration. We present the effect of boron on the crystallinity of CoFeB into two categories: the critical boron concentration (5 ~ 6%) at which CoFeB crystallizes and the effect of remaining boron (0 ~ 5%) in the crystallized CoFeB. And the trends of the saturation magnetization, exchange stiffness, exchange length, domain wall energy and Gilbert damping constant according to the boron concentration are provided. Abrupt variation of properties near the critical boron concentration (5 ~ 6%) and a noticeable change in the crystallized CoFeB (0 ~ 5%) are confirmed, revealing a clear causal relationship with the structural analysis. These results propose that the crystallization, microstructure, and major magnetic properties of CoFeB are governed by the amount of boron, and emphasize the need for delicate control of boron concentration.

## Introduction

CoFeB has been established as a representative material for spin-based devices after the discovery of huge tunneling magnetoresistance (TMR)^1^ and interfacial perpendicular magnetic anisotropy (iPMA)^2^, enabling higher integration, higher thermal stability (Δ), and lower switching current density (*J*_sw_). Among them, the two core properties, TMR and iPMA, are implemented through the fine control of crystallinity of CoFeB. Since TMR is originated from the in-plane momentum conservation of the spins in the process of tunneling through MgO barrier, the half-metallic nature of the Δ_1_ band of the ferromagnetic material and the band dependent transparency of the MgO layer play a key role^3^. For this, excellent crystallinity of the two materials and well-matched growth of CoFeB according to MgO layer are required^4^. Even in the case of iPMA, MgO-matched crystallization of CoFeB is also needed because iPMA is originated from the formation of local bonding or hybridization between ferromagnetic and oxygen atoms^2,5–9^.

For the MgO-matched growth of CoFeB, a crystallization process of amorphous CoFeB through annealing was introduced^2,9–11^. And the important roles of boron in this process have been revealed through many previous studies^8–19^. Before annealing, boron acts as a glass former to form an amorphous phase of CoFeB to suppresses the formation of crystal structures other than *bcc* and to cultivates the highly (100) oriented MgO layer^9–12^. During annealing, boron intensively diffuses in the opposite direction of the MgO layer having high defect formation energy and escapes to an absorbing layer such as tantalum, thereby allowing CoFe(B) to grow along the *bcc* MgO layer^8,13–16^.

Among these roles of boron, the decrease in the amount of boron in CoFeB due to inter-layer diffusion and subsequent crystallization is the most crucial mechanism for the implementation of MgO-matched *bcc* crystal of CoFeB. However, most of the studies on this subject have been limited to observing the distribution of boron or comparing relative concentrations using energy dispersive x-ray spectroscopy (EDS), secondary ion mass spectrometry (SIMS), electron energy loss spectroscopy (EELS) or x-ray photoelectron spectroscopy (XPS)^12,16–19^. There is no study confirming the specific boron concentration at which crystallization of CoFeB occurs, and the effects of remaining boron in CoFeB on the crystallinity after annealing. In this context, here we set boron concentration as a control parameter and evaluated the microstructure and magnetic properties of CoFeB. The microstructure of the CoFeB layer according to the boron concentration was confirmed through high resolution transmission electron microscopy (HRTEM) and x-ray diffraction (XRD). And the trends of the saturation magnetization (*M*_S_), exchange stiffness constant (*A*_ex_), exchange length (*l*_ex_), domain wall energy (*σ*_DW_) and Gilbert damping constant (*α*) were obtained by Brillouin light scattering (BLS) and ferromagnetic resonance (FMR).

Three main results are drawn in this study. The first, we present an abrupt crystallization of CoFeB when boron concentration is smaller than certain amount. And the second is a detailed evaluation of the microstructure of crystallized CoFeB according to the boron concentration. The third is the clear causal relation of microstructural characteristics on magnetic properties (*M*_S_, *A*_ex_, *l*_ex_, *σ*_DW_, and *α*). The above three results directly and strongly confirm the proposition that the crystallization, microstructure, and magnetic properties of CoFeB is governed by the internal boron concentration. And this conclusion emphasizes the need for delicate control of boron concentration to secure favorable magnetic properties.

## Materials and methods

The samples were deposited by the co-sputtering method, and the structure was constructed as follows [Si substrate/buffer/MgO (1)/CoFeB (18, 22)/MgO (1)/capping] (unit of number is nm). The composition of CoFeB was set using the deposition ratio according to the DC power of each CoFe and CoFeB target during co-sputtering. The composition ratio of CoFe was fixed as 10:90, and only the fraction of boron was changed as (Co_10_Fe_90_)_100−x_B_x_. And all experiments were carried out using as-deposited samples since we focused on the evaluation of the crystallinity of CoFeB according to the average boron concentration in the CoFeB layer set through the deposition ratio. Therefore, all boron composition is nominal values in our study. In addition, in order to suppress the inter-layer diffusion of boron after deposition, the MgO/CoFeB/MgO sandwich structure was constructed using the characteristics of MgO having high defect formation energy for boron^8^. The thickness of CoFeB was set to 22 nm suitable for the crystallinity analysis of the layer and interface through cross-sectional TEM image and fast Fourier Transform (FFT), and for evaluating *A*_ex_, which is deeply related to the structural characteristics. 18-nm thick samples were prepared to verify the magnetic properties for 5 ~ 8% boron concentration samples.

For BLS measurement, (3 + 3) Fabry–Perot interferometer was employed to obtain spin wave (SW) spectra^19^. As the light source, a single longitudinal mode laser of 532-nm wavelength with an output power of 300 mW was used. The light scattered with SW was acquired by the back scattering geometry, and the incident angle was fixed at 45 degrees corresponding to the magnon wave vector (*k*) of 16.70 µm^−1^. When acquiring SW spectra of all samples, the accumulation time was set as 2 h or more to secure sufficient signals. All measurements were carried out in ex-situ condition at room temperature. While measurement, a DC external magnetic field (from 0.02 to 0.90 T) was applied parallel to the film plane. Under these conditions, two kinds of SW modes were obtained: the surface or Damon-Eshbach (DE) mode^20^, and the bulk mode^21^. For more details, see Supplementary information sec. 1.

In addition to the BLS measurement, we carried out FMR experiments to obtain *α*, and to confirm the trend of *M*_S_ obtained by BLS. For FMR experiments, the Quantum Design Physical Property Measurement System and cryoFMR of NanOsc Instruments was employed. In this system, the sample was mounted on a coplanar waveguide (CPW) connected to an RF generator (input) and an RF diode (output). The measurement was carried out by sweeping the external magnetic field (0.5 mT steps) applied to parallel to the film plane, while fixing the frequency of RF. As a result, the external magnetic field dependence of the FMR absorption was obtained. For more details, see Supplementary information sec. 2.

## Results and discussion

**Figure 1:**
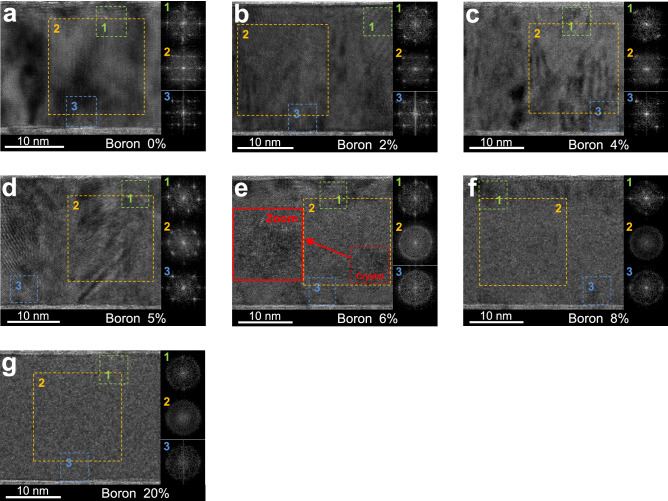
Transmission electron microscopy results. (**a**–**g**) cross-sectional TEM images for various boron concentration. And inset images (1–3) are results of the fast Fourier transform (FFT) process for square dashed selected areas. Inset 1, 3 for top and bottom interface regions (5 × 5 nm^2^) and inset 2 for the middle of layer region (16 × 16 nm^2^). Especially in (**e**), the locally crystallized area (red dash square) was drawn enlarged to the left (red square).

**Figure 2:**
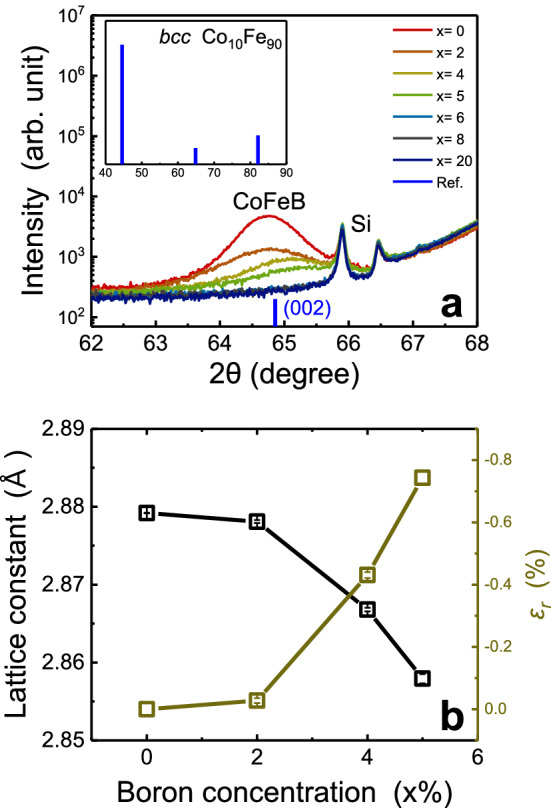
*θ*−*2θ* scan x-ray diffraction (XRD) results. (**a**) XRD spectra for various boron concentration samples. Inset in (**a**) indicates the peak position and intensity of literature *bcc* Co_10_Fe_90_ (Ref.^22^). (**b**) The obtained lattice constant and relative compressive strain according to boron addition.

Figure 1 shows the cross-sectional TEM images of the MgO(1)/CoFeB(22)/MgO(1) for various boron concentrations. In each figure, the insets 1, 2 and 3 are FFT results (performed by ImageJ program) of the selected areas of the top (1, green), middle (2, orange), and bottom (3, blue) region of CoFeB layer, respectively. The size of the selected square area of the interfaces and middle of layer are 5 × 5 nm^2^ and 16 × 16 nm^2^, respectively. Figure 2a and b show *θ-2θ* scan XRD spectra and peak analysis results of CoFeB according to boron concentration. The lattice constant for each boron concentration was determined through the center position of the XRD peak (black squares), and the relative compressive strain (*ε*_r_) was determined with the change of lattice constant from 0% boron sample (${\varepsilon }_{r}=\frac{{a}_{x}-{a}_{0} }{{a}_{0}}\times 100$) according to *x*% boron addition (khaki squares). Over 6% boron concentration, the lattice constant and strain could not be obtained because of disappeared XRD peak intensity as shown in Fig. 2a.

To begin with, we describe the critical boron concentration at which the amorphous CoFeB layer crystallizes. The cross-sectional TEM images and FFT patterns for the middle of layer of CoFeB (Fig. 1a–g, inset 2) show the shape of textured poly-crystal/defined FFT spots up to 5% respectively, and untextured structure/ring shaped FFT pattern from 6% of boron^7^. In addition, in the XRD result shown in Fig. 2a, the *bcc* (002) peak of Co_10_Fe_90_ appears only at the boron concentration of 0 ~ 5%, and no peak appears above 6%^12,23,24^. These two results clearly indicate that amorphous CoFeB crystallizes abruptly from 5% boron concentration. Accordingly, the 5% boron concentration can be understood as a critical concentration for the crystallization of CoFeB, and based on this, the entire boron concentration region can be divided into two sections in which CoFeB is crystalline (≤ 5%) and amorphous (≥ 6%).

It must be mentioned that weak FFT spots are located in the ring-shaped FFT pattern (Fig. 1e, inset 2), and small locally crystallized region is formed in the amorphous section in the middle of layer (red lines in Fig. 1e). This can also be understood on the basis of the previously defined critical crystallization boron concentration. Since 6% is near the threshold concentration, the crystallization of CoFeB can be easily occurred in the less boron regions formed by boron diffusion due to external stimulation. In this case, the main factor of the stimulation can be estimated as the kinetic energy transmitted by sputtered atoms during the deposition process. And at the boron concentrations of 8% and more in which more boron is added from the crystallization point, the middle regions of layer are entirely amorphized without local crystalline regions. Clear rings without any weak FFT spots can be seen in the insets 2 of Fig. 1f,g, which supports this analysis.

In addition, several crystallized regions are located at the interfaces with upper MgO (about 3 nm) for the 6 ~ 8% boron samples in the amorphous sections (Fig. 1e,f, inset 1). On the other hand, relatively less localized crystal regions are formed at the interfaces with lower MgO (Fig. 1e,f, inset 3). The interfacial crystallization formed to different degrees at the top and bottom interfaces can also be understood as the formation of less boron regions. But in this case, the formation of the less boron region is ascribed to the stimulus applied during the deposition of upper MgO^12^, and boron absorbing of the upper MgO layer can also be considered as another cause^18^. Subsequently, interfacial crystallinities are not formed at 20% boron concentration. This is due to the increase of the average boron concentration in the CoFeB layer, making the formation of less boron regions difficult.

We have so far confirmed that there is a critical boron concentration at which CoFeB is crystallized, and that locally crystallized regions can be formed in the middle of the CoFeB layer or at the interface with adjacent layers by the formation of less boron regions via stimulation during deposition.

Next, we describe the microstructural characteristics according to the boron concentration in the region where CoFeB is crystalline (≤ 5%). In this concentration range, overall crystallinity of CoFeB is maintained, but remarkable changes in microstructure are shown with addition of boron. Four major changes can be observed with increasing boron concentration.

First, the change of lattice spacing according to boron concentration is confirmed. In Fig. 2b, a large decrease in the lattice constant with the addition of boron from 0 to 5% is confirmed. The extent of this change can be confirmed through the magnitude of the relative compressive strain values calculated through the change rate from the 0% sample. It is understood that this change in lattice constant and the relative compressive strain are due to the void action of boron in the *bcc* CoFe lattice.

Second, notable grain size reduction and shape change are observed (Fig. 1a–d). Between 0 and 2%, it is observed that the grain shape changes from particle to columnar structure^25^, and the size of the grain becomes much smaller^26^. On the other hand, at the concentration of 4 ~ 5%, the gran shape becomes closer to the columnar shape, but no significant change in grain size is observed. These grain characteristics can be understood that the dominant distribution type of boron changes from grain boundary segregation to lattice penetration before and after 2%, together with the XRD analysis result showing a large change in lattice constant at 4 ~ 5% concentration (Fig. 2b).

Third, when the boron concentration approaches near the critical point of phase transformation (4 ~ 5%), a locally formed amorphous phases in the cross-sectional TEM images are observed (Fig. 1c,d). And the FFT patterns in this region show tails along the ring shape seen in the amorphous samples (Fig. 1c,d,e–g, inset 2). This can be interpreted as the weakening of the lattice spacing regularity as the boron concentration approaches the critical point of phase transformation^25^. Accordingly, local amorphization proceeded in the region where more boron has been accumulated. The XRD result, which shows an abrupt change in lattice constant and compressive strain at 4 ~ 5% concentration supports above analysis (Fig. 2b)^25^. Fourth, those disturbance can also be observed at the interface with MgO. It can be seen that the epitaxy with MgO is remarkably reduced in 4 ~ 5% samples unlike 0 ~ 2%, due to the reduction of the grain sizes and broaden lattice spacing distribution (Fig. 1a–d).

In this way, we confirmed that even in crystalline CoFeB, remaining boron can cause crystallinity disturbance such as decrease in lattice constant, decrease of the grain size, grain shape change, broaden lattice spacing distribution, the formation of local amorphous regions, and a consequent degradation of MgO epitaxy. These results suggest that the amount of remaining boron after crystallization must be optimized to secure favorable microstructure characteristic.

**Figure 3:**
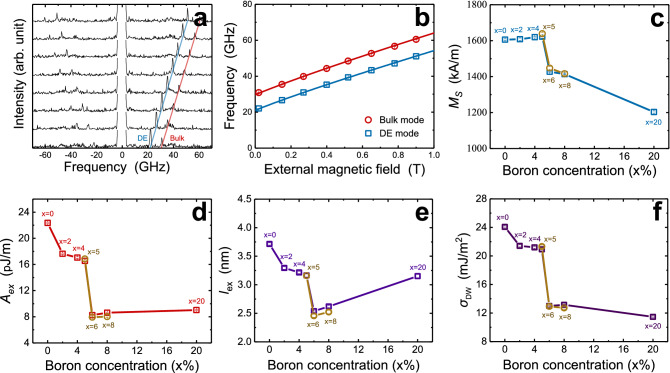
Brillouin light scattering (BLS) results. (**a**, **b**) Magnetic field dependence of anti-Stokes mode peaks and spin wave frequencies. And boron concentration dependences of (**c**) the saturation magnetization (*M*_S_), (**d**) exchange stiffness constant (*A*_ex_) obtained by BLS, and (**e**) the exchange length (*l*_ex_) and (**f**) domain wall energy (*σ*_DW_) calculated with the obtained *M*_S_ and *A*_ex_ values. The CoFeB composition of all sample is (Co_10_Fe_90_)_100−x_B_x_. All brown disk points in (**c**–**f**) mean the parameter values obtained with 18-nm thick CoFeB layer of the same composition ratio.

**Figure 4:**
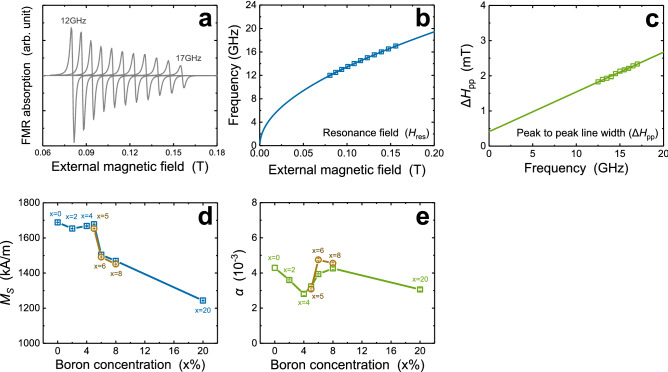
Ferromagnetic resonance (FMR) results. (**a**) external magnetic field dependence of FMR absorption and RF frequency dependence of (**b**) resonance field (*H*_res_) and (**c**) peak to peak line width (Δ*H*_pp_). Boron concentration dependence of the (**d**) saturation magnetization (*M*_S_) and (**e**) Gilbert damping constant (*α*) for (Co_10_Fe_90_)_100−x_B_x_. All brown disk points in (**d**, **e**) mean the parameter values obtained with 18 nm CoFeB of the same composition ratio.

The magnetic properties according to the boron concentration described in this section have a clear causal and reinforcing relationship with the results of the microstructure analysis. Figure 3a and b shows the external magnetic field dependence of the DE and Bulk mode frequencies obtained through BLS. Equations for each mode were applied to the experimental data to evaluate *M*_S_ and *A*_ex_. Used equations and detailed analysis procedures such as explanation of formulas and setting of constants for fitting are described in Supplementary Information sec. 1. Figure 3c shows the dependence of *M*_S_ on the boron concentration obtained by BLS measurements. The blue square points represent the result of 22 nm CoFeB samples, and the brown disk points represent the result of the 18 nm samples. In the range of 0 ~ 5% boron in which CoFeB is crystallized, almost the same value of *M*_S_ is shown. This is because of the little effect of boron on the 3*d* band of CoFe since the crystallinity of CoFeB is maintained. In other words, since the *sp*-*d* band hybridization of the two materials by amorphization did not proceed, the electrons in the *p*-band of boron were not transferred to the *d*-band of CoFe due to the large energy difference between the two bands^22^. Next, in the boron 5 ~ 6% section where the CoFeB layer loses its crystallinity, sharp decrease of *M*_S_ is observed by about 200 kA/m. This ascribe to the amorphization of CoFeB and consequent changes of the distribution or filling in the 3*d* band of CoFe by *sp-d* hybridization^22^. Above 6%, linear decrease of *M*_S_ is observed with the addition of boron, which has been confirmed in previous studies as a result of a decrease in magnetic moment due to more *sp-d* hybridization^22,26^. The boron concentration dependence of *M*_S_ obtained through BLS can be reconfirmed by result of FMR (Fig. 4d).

Figure 3d shows the trend of *A*_ex_ according to the boron concentration evaluated by BLS. The red squares represent the result of 22 nm CoFeB samples, and the result of 18 nm samples are also depicted by the brown disks in order to confirm the abrupt change of *A*_ex_ around boron 5 ~ 6%. *A*_ex_, by definition, means the strength of exchange interactions with the nearest atoms, and is closely related to the short-range crystallinity such as number of neighboring atoms, crystal structure, lattice constant, and the strength of internal atomic bonds. However, since evaluated *A*_ex_ value is an average value for all spins within the measurement range, it is obvious that microstructural features (grain boundary, grain size, grain shape) affect the values. Accordingly, in this paper, the trend of *A*_ex_ is interpreted based on the structural analysis at the microstructure level. In the 0 ~ 5% concentration where CoFeB is crystalline, decreasing trend of *A*_ex_ is observed according to the addition of boron. In particular, the significant decrease of *A*_ex_ is confirmed at 0 ~ 2% (~ 5 pJ/m), which can be understood as a result of the decrease in grain size and the change of grain shape from particle to columnar structure^24–28^. As the grain size decreases, the density of the grain boundary increases, so it is naturally accepted that the number of neighboring atoms or the strength of bonds decreases in the region made up of imperfect bonds. Also, decrease in the strength of atomic bonds on the shorter side of columnar grain is also considered to be one possible cause. At 2 ~ 5% concentration, slight decrease of *A*_ex_ is observed (~ 1 pJ/m), which also seems to be related to the weakening of atomic bonds indicated by the strain, widened distribution of lattice spacing, and decrease of the nearest neighborhood number of ferromagnetic atoms. In the 5 ~ 6% section where CoFeB loses crystallinity, a sharp decrease of *A*_ex_ is confirmed (~ 8 pJ/m), which can be understood as a result of the disorganization of the lattice due to amorphization and consequent decrease in the number of neighboring atoms, and the increase in the average lattice spacing. Then, when boron concentration is added to amorphous CoFeB (≥ 6%), almost the same value of *A*_ex_ is maintained, which ascribes to little effect of the added boron on the strength of atomic bonds or number of neighboring atoms in amorphized structure.

In addition, the exchange length (*l*_ex_) and domain wall energy (*σ*_DW_) calculated using obtained *M*_S_ and *A*_ex_ is shown in Fig. 3e,f. The *l*_ex_ $\left(\equiv \sqrt{\frac{{A}_{ex}}{{K}_{eff}}}=\sqrt{\frac{2{A}_{ex}}{{\mu }_{0}{M}_{S}^{2}}}\right)$ and *σ*_DW_ $\left(\equiv 4\sqrt{{A}_{ex}{K}_{eff}}=4\sqrt{\frac{1}{2}{A}_{ex}{\mu }_{0}{M}_{S}^{2}}\right)$ were calculated with the effective anisotropy energy density (*K*_eff_) considering only demagnetization energy term $\left({K}_{eff}=\frac{1}{2}{\mu }_{0}{M}_{S}^{2}\right)$. This is valid because the thickness of this sample series is 18, 22 nm, the contribution of interface anisotropy energy density (*K*_i_) to *K*_eff_ is very small compared to demagnetization energy, and thus can be neglected^29^. Between boron 0 ~ 6%, decreasing trend of *l*_ex_ is observed following the trend of *A*_ex_. This ascribes to almost the same *M*_S_ and decreasing *A*_ex_ in this range as shown in (Fig. 3c,d). Conversely, after the structure becomes amorphous (more than 6%), linear increase of *l*_ex_ is observed because of linearly decreasing *M*_S_ and almost the same *A*_ex_ in that concentration range^30^. On the other hand, the trend of *σ*_DW_ mainly follows the trend of *A*_ex_. In conclusion, these two results suggest that *l*_ex_ and *σ*_DW_ can be varied greatly depending on the crystallization and crystalline characteristics according to the boron concentration. And these results should be considered as a result for supplementing the limitations of the macro-spin model using the CoFeB layer, and as a condition for suppressing sub-volume excitation, multi-domain switching^31,32^.

Finally, we describe the results of FMR analysis. As shown in Fig. 4a, the external magnetic field dependence of the FMR absorption was obtained in the 12–17 GHz RF frequency range with 0.5 GHz steps. And the trends of resonance field (*H*_res_) and peak to peak line width (Δ*H*_pp_) according to frequency were obtained, and as a result of fitting them using equations (in Supplementary Information sec. 1), *M*_S_ and *α* values were evaluated according to boron concentration. Figure 4b and c are examples of fitting the obtained experimental value using each equation. In the example, experimental values are drawn as squares, and fitting curves as solid lines. In this paragraph, a discussion focusing on *α* is presented since the result of *M*_S_ was already mentioned previously in the context of the verification of trends obtained through BLS.

The trend of the *α* according to the boron concentration is shown in Fig. 4e. The green squares represent the result of 22 nm CoFeB samples, and the brown disks represent the result of 18 nm samples. To begin with, even in the crystalline CoFeB region, the *α* value is small compared to the results of other previous studies with similar CoFeB thickness because of the Fe-rich composition of CoFeB^24,27,33,34^. Up to 4% of boron, decreasing trend of *α* is observed with the increasing boron concentration. The tendency of *α* to decrease with decreasing crystallinity, confirmed from previous studies, can be understood as the cause of this trend^27,35,36^. After that, in the 4 ~ 8% boron concentration range, trend of increasing *α* is observed. Two possible causes for this result can be suggested. First is the lattice spacing distribution. In the above structural analysis of the 4 ~ 5% sample, the broaden lattice spacing distribution of CoFeB crystals was confirmed, and this characteristic can act similar to the case of a/c ratio^37^. In other words, such broadening can act as a scattering source for the SW and/or energy dissipation pass, so it can be a cause of the increase in *α*. The second is the increase in the line width of FMR absorption (see Supplementary information Sec. 2) due to the amorphous-crystalline mixed state in that boron concentration range (4 ~ 8%). we estimate that the increase in *α* also can be affected by anisotropy distribution from the amorphous-crystalline mixture in the middle of layer (4 ~ 5%) or non-uniformity between the middle of layer and the interfaces (6 ~ 8%)^38,39^. At 20% where CoFeB shows a homogeneous amorphous phase, and *α* decreases to the lowest value, which supports above understanding.

In this way, we confirmed the trends of various magnetic properties according to boron concentration. Among them, especially *A*_ex_ is an important property in the magnetic random access memory (MRAM) and skyrmion-based devices studies. In the MRAM field, it is well known that sufficient *A*_ex_ value should be secured to increase the energy barrier for sub-volume excitation thus to obtain proper effective thermal stability^31^. And large *A*_ex_ can also contribute the reduction of the *J*_sw_ and distribution of switching probability by suppressing the effect of formation of multi-domains and effect of non-collinear spin configuration by DMI^31,32,40^. For the skyrmion-based devices, high DMI and low *σ*_DW_ are required to form a stable Néel-type skyrmion^41^. But it is difficult to implement such high DMI in a polycrystal system, so lowering *σ*_DW_ could be a better approach. In this context, this result showing sensitive change of *A*_ex_ depending on crystal quality and abrupt decrease of *A*_ex_ after amorphization is worth considering in theoretical calculations and optimization process for devices using CoFeB materials.

## Conclusion

In summary, we confirmed the structural-magnetic properties of as-deposited CoFeB according to the boron concentration in the MgO/CoFeB/MgO structure, which partially suppresses the inter-layer diffusion of boron from the CoFeB layer. Our experimental findings can be divided into three categories. First is that the crystallization of CoFeB proceeds below 5% boron. And it was shown that even at higher boron concentrations (≥ 6%), local crystallization can occur due to the formation of less boron regions by stimulation applied during deposition. In particular, this process was more likely to occur at the interface with other layers. Second is the weakening of the long-range crystallinity, including compressive strain, grain size reduction, grain shape change, formation of local amorphous phases, and reduction of MgO epitaxy with the addition of boron concentration in the crystallized CoFeB (≤ 5%) samples. Third is the trends of *M*_S_, *A*_ex_, *l*_ex_, *σ*_DW_, and *α* due to *sp*-*d* hybridization by boron influx, reduction of long-range crystallinity, lattice spacing distribution, and non-uniform crystal structure at the layer and interface. And the trends of properties showed a clear causal and reinforcing relationship with the microstructural analysis. Our results can be considered as quantitative criteria for crystallization, crystalline characteristics, and magnetic properties according to boron concentration in a representative process of extracting boron through annealing and crystallizing CoFeB.

## Supplementary Information

Supplementary Information.

## Data Availability

The data that support the findings of this study are available from the corresponding author upon reasonable request.
