# Supplementary material for: Control of crystallization and magnetic properties of CoFeB by boron concentration
Source: Sci Rep. 2022 Mar 16;12:4549. doi: 10.1038/s41598-022-08407-6 (PMC8927602; doi:10.1038/s41598-022-08407-6)
Supplement: Supplementary file 1 — Supplementary Information. [file 41598_2022_8407_MOESM1_ESM.pdf]

Supplementary Information

Control of crystallization and magnetic properties of CoFeB by boron concentration

Jun-Su Kim[a], Gukcheon Kim[b], Jinwon Jung[b], Kuyoul Jung[b], Jaehun Cho[c], Woo-Yeong Kim[d], Chun-Yeol You[a,*]

[a] *Department of Emerging Materials Science, DGIST, Daegu, Republic of Korea*

[b] *SK hynix Inc., Icheon, Republic of Korea*

[c] *Convergence Research Institute, DGIST, Daegu, Republic of Korea*

[d] *Department of Materials Science and Engineering, Korea University, Seoul, Republic of Korea*

*Correspondence : Chun-Yeol You (cyyou@dgist.ac.kr)

Section 1. Measurement and analysis process of Brillouin light scattering (BLS)

Subsection 1 -1. BLS measurement system.

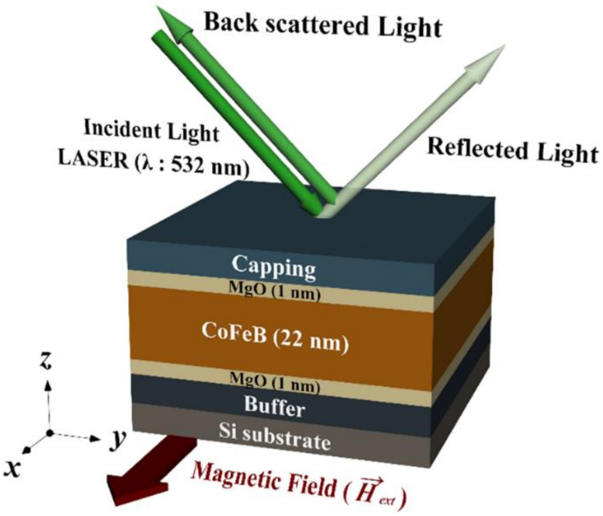

Fig. S1. BLS measurement system. Under a dc external magnetic field (H_{ext}), the frequency of the light after scattering with the DE and Bulk mode was obtained in back scattering geometry.

The scheme of the BLS measurement system is depicted in Fig. S1. In this system, a dc H_{ext} (0.2 to 9.0 kOe) was applied along x direction of the easy axis (in-plane). Subsequently, Damon-Eshbach (DE, surface) mode spin wave (SW) propagates toward $\pm y$ direction and a single node (n=1) bulk mode is formed in the z direction.[1,2] The measurement was performed using back scattering geometry with 532 nm wavelength (λ) incident laser light. The angle (θ) between the incident light and the sample normal was fixed at 45 degree corresponding to 0.0167 nm^{-1} of the magnon wave vector (k) (Eq. (S1)). For more detail see reference.[3,4]

$$k = \frac{4\pi}{\lambda}\sin\theta \qquad \text{(S1)}$$

Subsection 1 -2. BLS analysis process and calculation of SW frequency-CoFeB thickness.

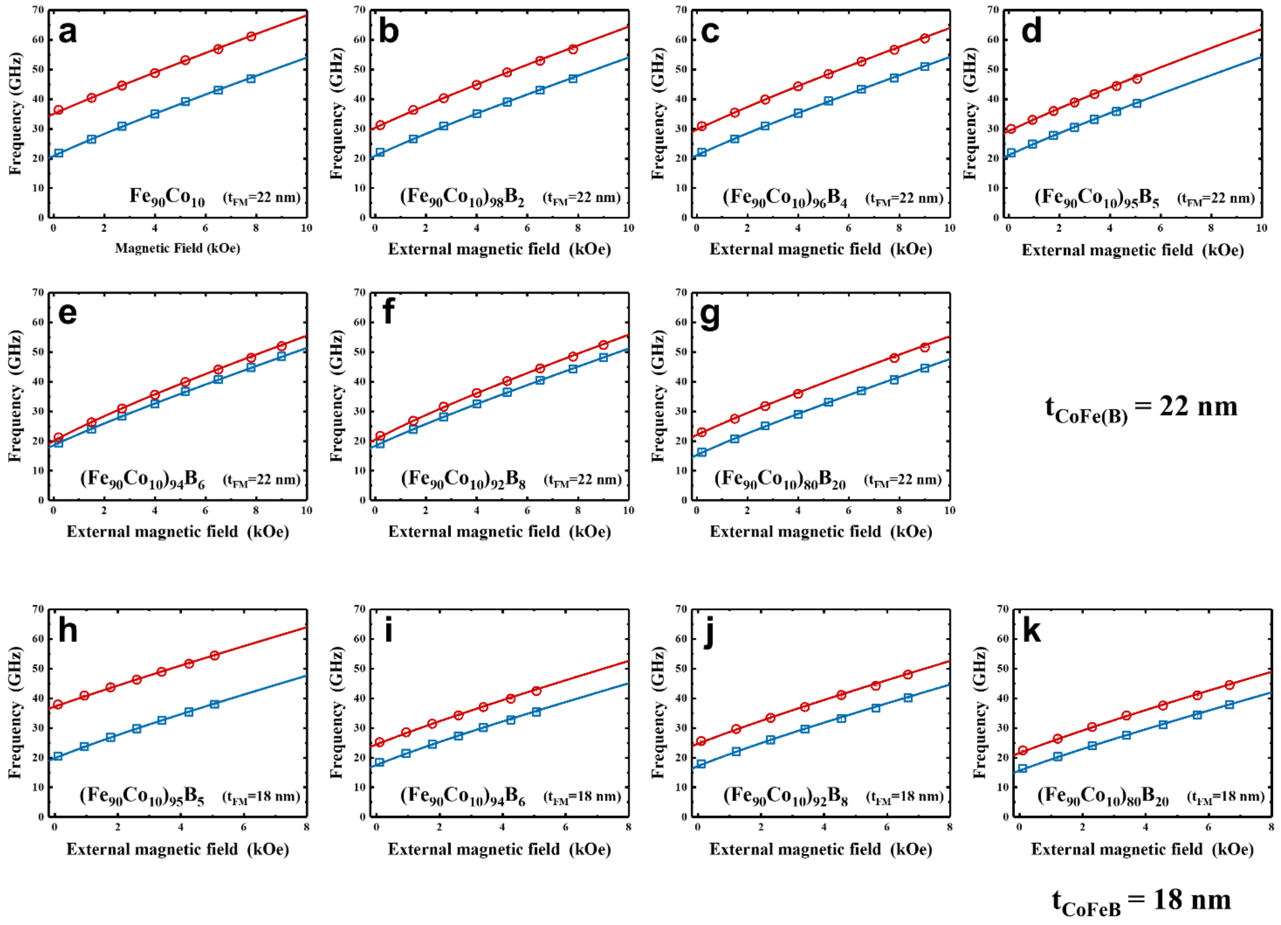

Fig. S2. BLS analysis back data. The external magnetic field dependence of the DE and bulk mode frequency of **(a-g)** 22 nm CoFe(B) and **(h-k)** 18 nm CoFeB by various boron concentration.

The trends of the DE and bulk mode frequencies according to the H_{ext} obtained through the BLS experiment is shown in Fig. S2. The saturation magnetization (M_S) and the exchange stiffness constant (A_{ex}) were evaluated by fitting the H_{ext} dependence of each modes' frequency using the DE, bulk mode equations (Eq. (S2-3)).[5]

$$f_{DE} \quad = \frac{\gamma}{2\pi}\left[H_{ext}(H_{ext}+\mu_0 M_S)+\left(\frac{\mu_0 M_S}{2}\right)^2\left(1-e^{-2kd}\right)\right]^{\frac{1}{2}} \tag{S2}$$

$$f_{Bulk} = \frac{\gamma}{2\pi}\left[\left(H_{ext}+\frac{2A_{ex}}{M_S}\left(k^2+\left(\frac{n\pi}{d}\right)^2\right)\right)\times\left(H_{ext}+\frac{2A_{ex}}{M_S}\left(k^2+\left(\frac{n\pi}{d}\right)^2\right)+\mu_0 M_S\right)\right]^{\frac{1}{2}} \tag{S3}$$

In Eq. (S2), f_{DE} (GHz) is the SW frequency of DE mode. In this case, the unit of H_{ext} and M_S is kOe and kA/m, respectively. γ (GHz/T) is the gyromagnetic ratio defined as $\gamma = \frac{ge}{2m}$, and consists of *g*-factor (*g*), charge of electron (*e*), and mass of electron (*m*). Value of γ in the equation was fixed to 180.26 GHz/T by setting the *g*-factor to 2.05 based on reference.[6] Other than that, *k* (nm^{-1}) is the magnon wave vector, and *d* (nm) is the thickness of the ferromagnetic material. Eq. (S3) also has the same constant configuration as above, but in particular, the node number (*n*), M_S, and A_{ex} are included here. Since we observed the bulk mode of the 1st node experimentally, *n* was set to 1. For M_S in the formula, the value obtained through DE mode analysis was substituted. Through these processes, M_S and A_{ex} were evaluated.

It must be mentioned that frequencies of the DE-bulk modes appear close to each other in the 6, 8% boron concentration 22-nm samples as presented in Fig. S2e-f . In order to eliminate mode confusion, the SW frequency of each mode was obtained again with 18-nm CoFeB samples of the same structure and same CoFeB composition. And the result is shown in Fig. S3.

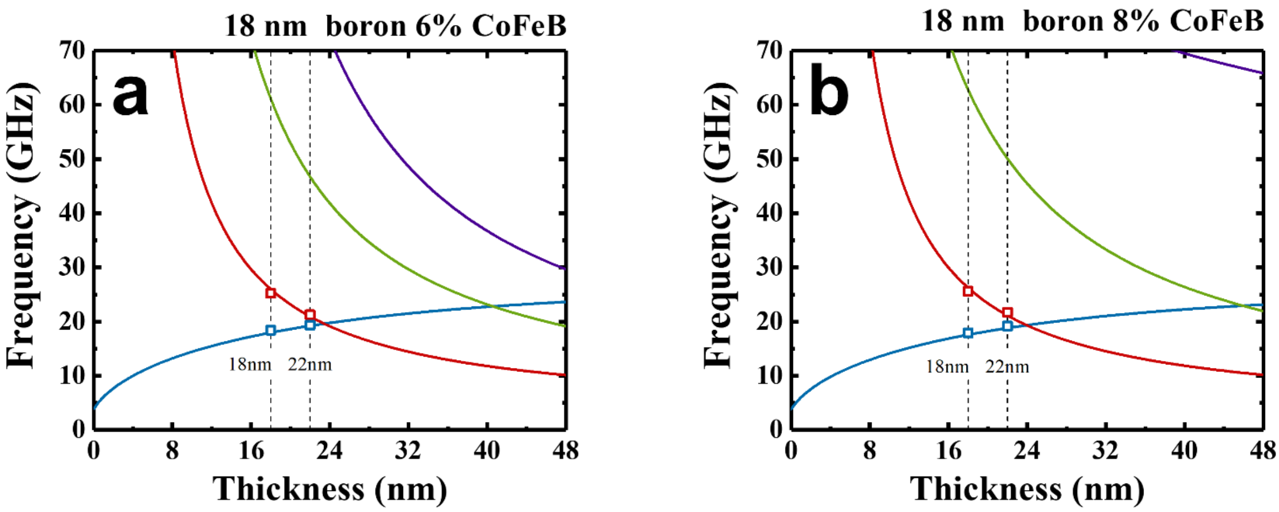

Fig. S3. Calculated SW frequency for CoFeB thickness. Calculated SW frequency of DE and bulk mode with obtained M_S and A_{ex} from 18 nm CoFeB with **(a)** 6% and **(b)** 8% boron concentration. The blue line represents the DE mode, and the red, green, purple lines represent the bulk mode (n=1, 2, 3), respectively. The blue and red square symbols are the DE, bulk mode frequencies of 18 and 22 nm CoFeB obtained through BLS experiments.

Fig. S3 shows the frequency - CoFeB thickness trends calculated with the physical properties measured from 18-nm thick CoFeB samples having composition of $(Co_{10}Fe_{90})_{94}B_6$ and $(Co_{10}Fe_{90})_{92}B_8$. Calculation was performed to distinguish two frequency of SW modes (DE, bulk) of 22-nm samples. This calculation was preformed based on the previous results that as the thickness of the magnetic layer decreases, the frequency of DE mode decreases and the frequency of bulk mode increases.[7,8] And the results show that the frequency of DE, bulk modes are close to each other but not inverted in 22-nm thick CoFeB samples. Through this result, the reliability of M_S and A_{ex} found in the 22-nm sample can be secured. And it can be explained that the change in the frequency trends of DE and bulk modes for each boron concentration is clearly due to the change in physical properties.

Section 2. Measurement and analysis process of ferromagnetic resonance (FMR)

Subsection 2 -1. FMR measurement system.

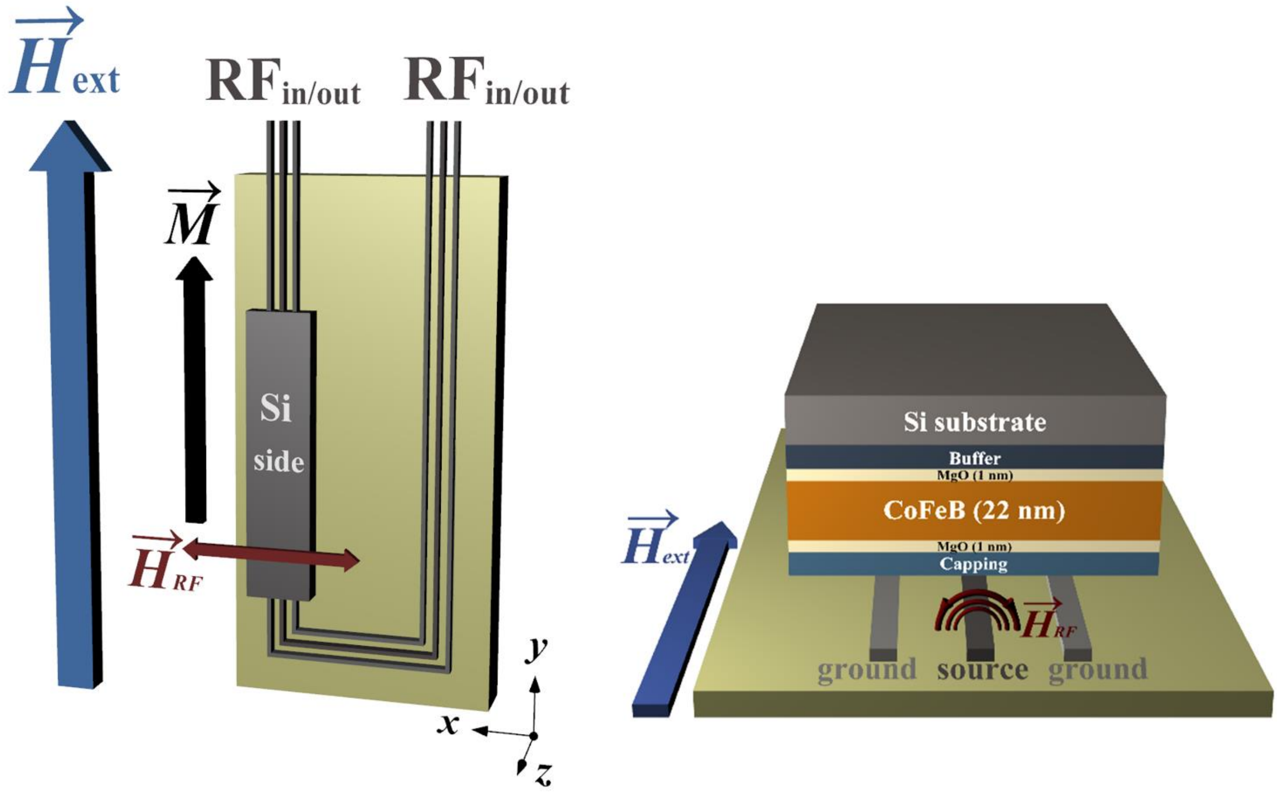

Fig. S4. FMR measurement system. The sample was mounted on the coplanar waveguide (CPW), And a dc H_{ext} and a RF magnetic field (H_{RF}) were applied, and the power absorption by FMR between the SW and the H_{RF} was measured according to the magnitude of the H_{ext}.

In FMR system shown in Fig. S4, the sample was mounted in contact with the coplanar waveguide (CPW), and the RF electric pulse was injected along source line of the CPW. Accordingly, a RF Oersted magnetic field (H_{RF}) was applied along $\pm x$ direction on the CPW plane.[8-11] Then, a dc external magnetic field (H_{ext}) was applied along y direction of the easy axis (in-plane), and the power absorption by FMR between H_{RF} and the uniform SW (k~0)[9,12,13] was measured according to the magnitude of the H_{ext} at each fixed frequency of RF.

Subsection 2 -2. FMR analysis process

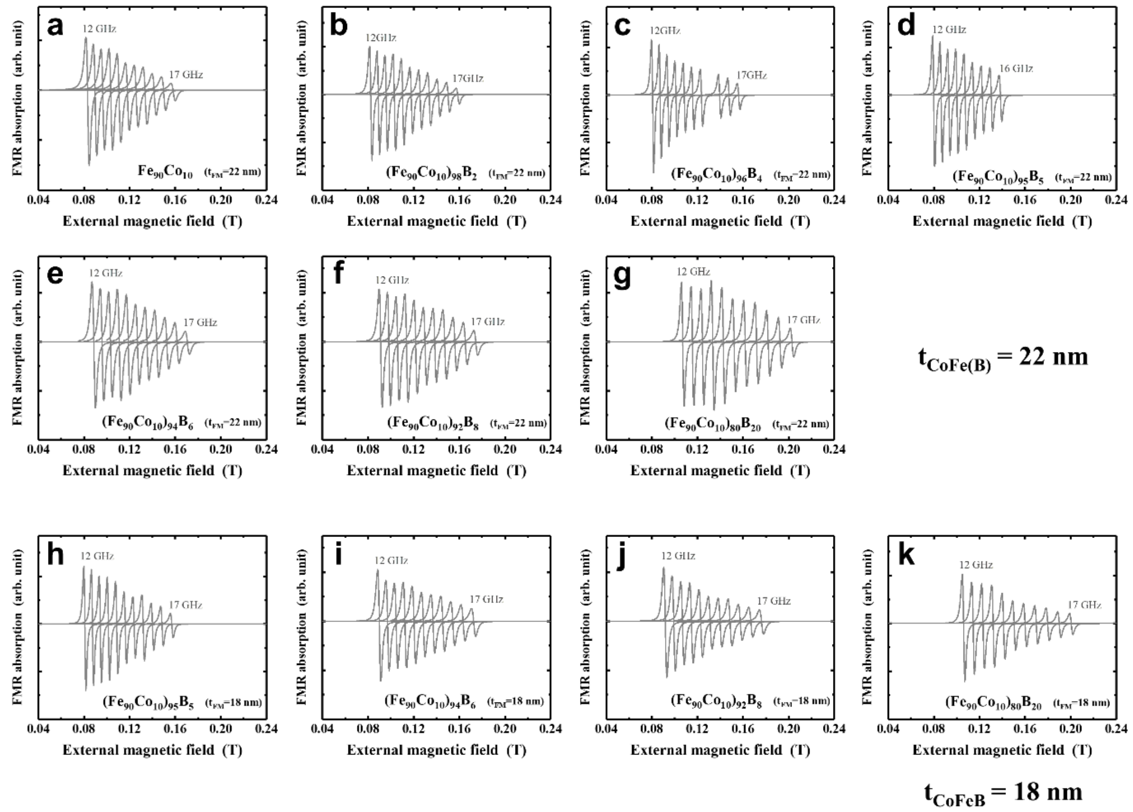

Fig. S5. External magnetic field dependence of FMR power absorption for **(a-g)** 22-nm CoFe(B) and **(h-k)** 18-nm CoFeB by various boron concentration.

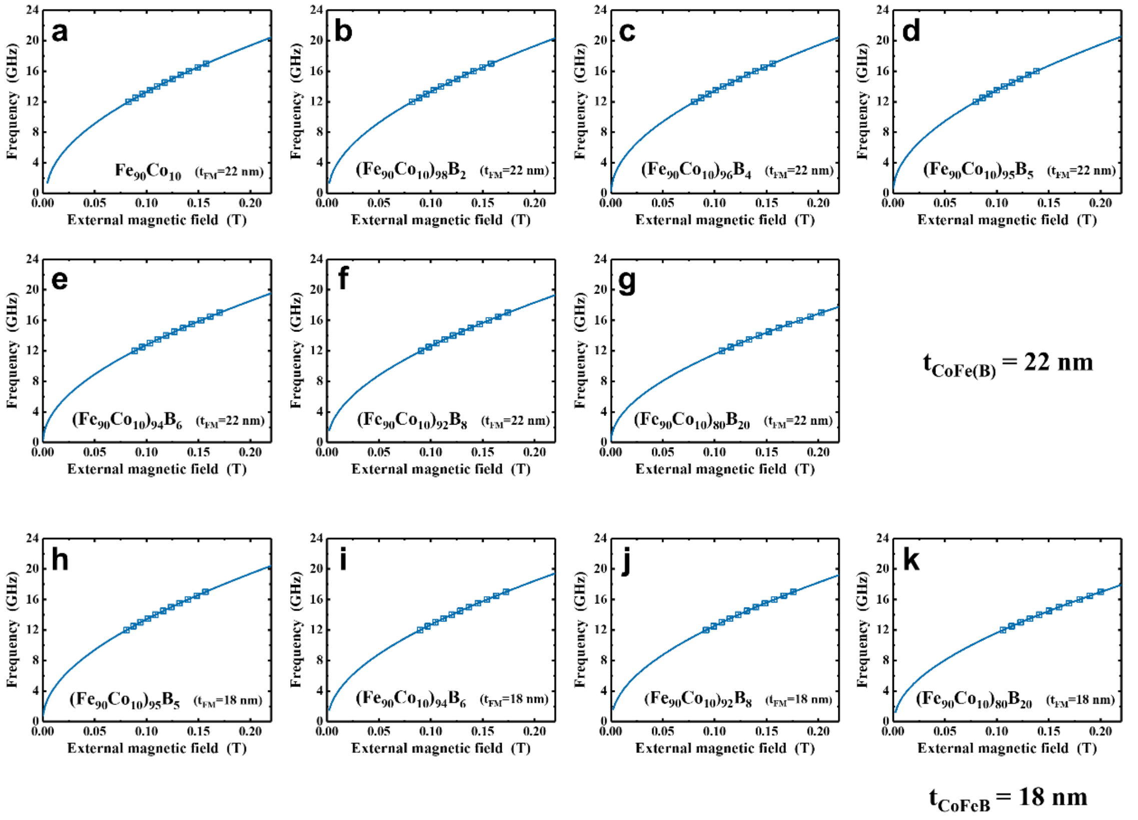

Fig. S6. RF frequency dependences of resonance field (H_{res}) for **(a-g)** 22-nm CoFe(B) and **(h-k)** 18-nm CoFeB by various boron concentration.

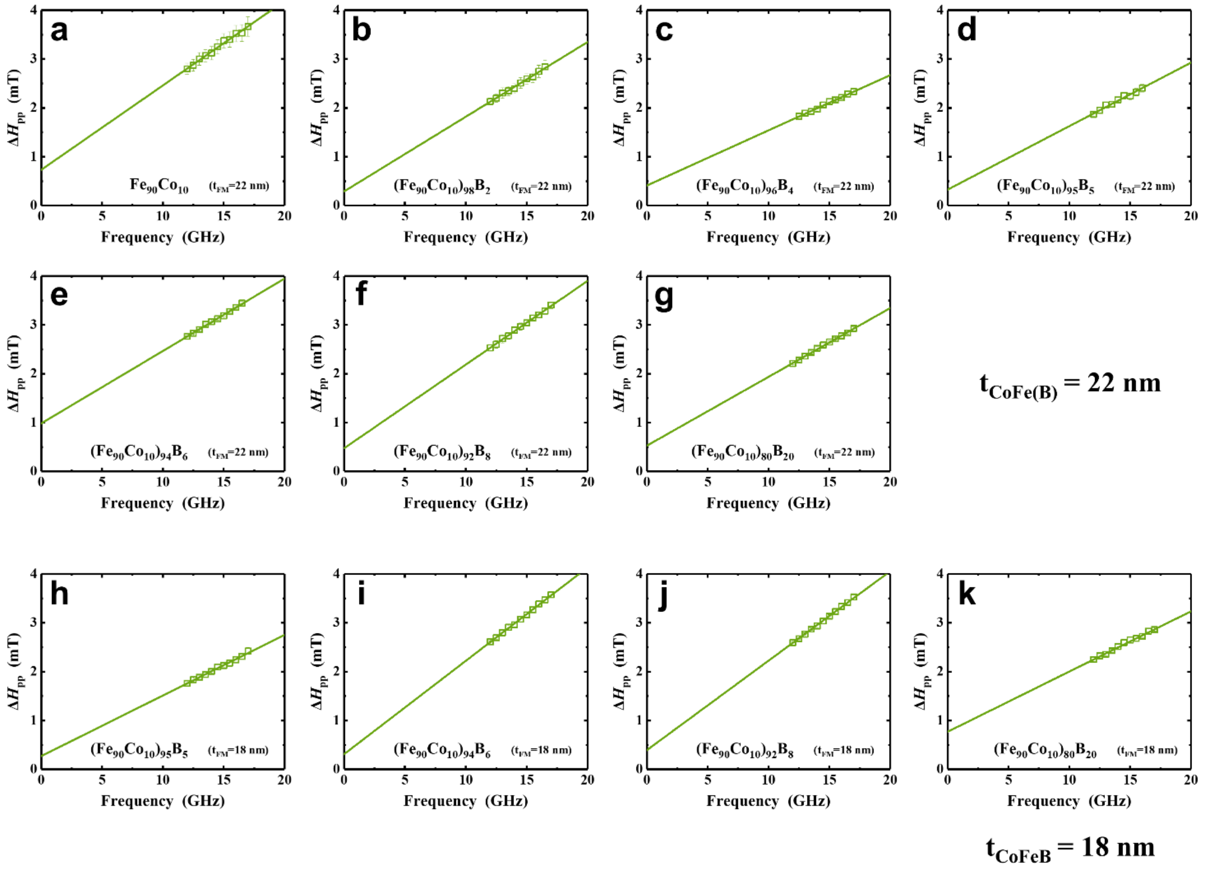

Fig. S7. RF frequency dependences of peak to peak line width (ΔH_{pp}) for **(a-g)** 22-nm CoFe(B) and **(h-k)** 18-nm CoFeB by various boron concentration.

FMR measurement was performed as external magnetic field sweep method with fixed RF frequency (12-17 GHz, step 0.5 GHz), and FMR absorption for the H_{ext} was obtained (Fig. S5). Through fitting process, the resonance magnetic field (H_{res}) and peak to peak line width (ΔH_{pp}) were obtained, and those were plotted according to the RF frequency. The RF frequency dependence of the obtained H_{res} and ΔH_{pp} (Fig. S6-7) were fitted by Eq. (S4) and (S5) to determine the M_{S} and α.[12-15]

The components of Eq. (S4) are the same as those of Eq. (S2-S3) above. However, the magnon wave vector (k) is set to 0 in the equation below since the FMR mode we observed is a uniform wave. In addition, the anisotropy field (H_{ani}) was set as a free fitting constant and showed a low significant figure that does not need to be considered. Eq. (S5) consists of α, extrinsic damping term ($\Delta H_{\mathrm{pp,0}}$), SW angular frequency (ω), and γ. In this analysis process, γ was fixed at 180.26 GHz/T as in the case of BLS.

$$f_{FMR} = \frac{\gamma}{2\pi}[(H_{ext} + H_{ani})(H_{ext} + H_{ani} + \mu_0 M_S)]^{\frac{1}{2}} \tag{S4}$$

$$\Delta H_{pp} = \Delta H_{pp,0} + \frac{2}{\sqrt{3}}\frac{\alpha\omega}{\gamma} \tag{S5}$$

References

1 Damon, R. W., Eshbach, J. R. Magnetostatic modes of a ferromagnet slab. *Journal of Physics Chemistry of Solids* **19**, 308-320 (1961).

2 Camley, R. E., Mills, D. L. Surface response of exchange-and dipolar-coupled ferromagnets: Application to light scattering from magnetic surfaces. *Physical Review B* **18**, 4821 (1978).

3 Cho, J. et al. Thickness dependence of the interfacial Dzyaloshinskii-Moriya interaction in inversion symmetry broken systems. *Nat. Commun.* **6**, 7635, doi:10.1038/ncomms8635 (2015).

4 Kim, D.-Y. et al. Quantitative accordance of Dzyaloshinskii-Moriya interaction between domain-wall and spin-wave dynamics. *Physical Review B* **100**, doi:10.1103/PhysRevB.100.224419 (2019).

5 Grünberg, P., Mayr, C. M., Vach, W., Grimsditch, M. Determination of magnetic parameters by means of brillouinscattering. Examples: Fe, Ni, Ni0. 8Fe0. 2. *Journal of Magnetism and Magnetic Materials* **28**, 319-325 (1982).

6 Devolder, T. et al. Damping of CoxFe80−xB20 ultrathin films with perpendicular magnetic anisotropy. *Applied Physics Letters* **102**, doi:10.1063/1.4775684 (2013).

7 Lenk, B., Eilers, G., Hamrle, J. & Münzenberg, M. Spin-wave population in nickel after femtosecond laser pulse excitation. *Physical Review B* **82**, doi:10.1103/PhysRevB.82.134443 (2010).

8 Ding, Y., Klemmer, T. J. & Crawford, T. M. A coplanar waveguide permeameter for studying high-frequency properties of soft magnetic materials. *Journal of Applied Physics* **96**, 2969-2972, doi:10.1063/1.1774242 (2004).

9 Yin, Y. et al. Tunable permalloy-based films for magnonic devices. *Physical Review B* **92**, doi:10.1103/PhysRevB.92.024427 (2015).

10 Yin, Y. et al. Ferromagnetic and Spin-Wave Resonance on Heavy-Metal-Doped Permalloy Films: Temperature Effects. *IEEE Magnetics Letters* **8**, 1-4, doi:10.1109/lmag.2016.2630663 (2017).

11 Behera, N., Guha, P., Pandya, D. K. & Chaudhary, S. Capping Layer (CL) Induced Antidamping in CL/Py/beta-W System (CL: Al, beta-Ta, Cu, beta-W). *ACS Appl. Mater. Interfaces* **9**, 31005-31017, doi:10.1021/acsami.7b06991 (2017).

12 Kittel, C. On the Theory of Ferromagnetic Resonance Absorption. *Physical Review* **73**, 155-161, doi:10.1103/PhysRev.73.155 (1948).

13 Herring, C. & Kittel, C. On the Theory of Spin Waves in Ferromagnetic Media. *Physical Review* **81**, 869-880, doi:10.1103/PhysRev.81.869 (1951).

14 O'Dell, R. A. et al. Post-Deposition Annealing Effects on Ferromagnetic CoFeB Thin Films. *IEEE Transactions on Magnetics* **54**, 1-7, doi:10.1109/tmag.2018.2845394 (2018).

15 Gilbert, T. L. Classics in Magnetics A Phenomenological Theory of Damping in Ferromagnetic Materials. *IEEE Transactions on Magnetics* **40**, 3443-3449, doi:10.1109/tmag.2004.836740 (2004).
